# The Imperative Use of Bacillus Consortium and Quercetin Contributes to Suppress Fusarium Wilt Disease by Direct Antagonism and Induced Resistance

**DOI:** 10.3390/microorganisms11102603

**Published:** 2023-10-21

**Authors:** Ali Hassan, Waheed Akram, Humaira Rizwana, Zill-e-Huma Aftab, Sana Hanif, Tehmina Anjum, Mona S. Alwahibi

**Affiliations:** 1 Department of Plant Pathology, Faculty of Agricultural Sciences, University of the Punjab, Lahore 54590, Pakistan; alihassanmoon267@gmail.com (A.H.); huma.dpp@pu.edu.pk (Z.-e.-H.A.); tehminaanjum@yahoo.com (T.A.); 2 Department of Botany and Microbiology, College of Science, King Saud University, Riyadh 11495, Saudi Arabia; hrizwana@ksu.edu.sa (H.R.); malwhibi@ksu.edu.sa (M.S.A.); 3 School of Agricultural, Environmental and Veterinary Sciences, Charles Sturt University, Wagga Wagga, NSW 2678, Australia; shanif@csu.edu.au

**Keywords:** Fusarium wilt, Bacillus, quercetin, antagonism, induced resistance

## Abstract

Fusarium wilt diseases severely influence the growth and productivity of numerous crop plants. The consortium of antagonistic rhizospheric Bacillus strains and quercetin were evaluated imperatively as a possible remedy to effectively manage the Fusarium wilt disease of tomato plants. The selection of Bacillus strains was made based on in-vitro antagonistic bioassays against *Fusarium oxysporum* f.sp. *lycoprsici* (FOL). Quercetin was selected after screening a library of phytochemicals during in-silico molecular docking analysis using tomato LysM receptor kinases “SILKY12” based on its dual role in symbiosis and plant defense responses. After the selection of test materials, pot trials were conducted where tomato plants were provided consortium of Bacillus strains as soil drenching and quercetin as a foliar spray in different concentrations. The combined application of consortium (*Bacillus velezensis* strain BS6, *Bacillus thuringiensis* strain BS7, *Bacillus fortis* strain BS9) and quercetin (1.0 mM) reduced the Fusarium wilt disease index up to 69%, also resulting in increased plant growth attributes. Likewise, the imperative application of the Bacillus consortium and quercetin (1.0 mM) significantly increased total phenolic contents and activities of the enzymes of the phenylpropanoid pathway. Non-targeted metabolomics analysis was performed to investigate the perturbation in metabolites. FOL pathogen negatively affected a range of metabolites including carbohydrates, amino acids, phenylpropanoids, and organic acids. Thereinto, combined treatment of Bacillus consortium and quercetin (1.0 mM) ameliorated the production of different metabolites in tomato plants. These findings prove the imperative use of Bacillus consortium and quercetin as an effective and sustainable remedy to manage Fusarium wilt disease of tomato plants and to promote the growth of tomato plants under pathogen stress conditions.

## 1. Introduction

Tomatoes are the second most commercially significant vegetable produced globally [1]. According to estimates, over 160 million tons of tomatoes were produced worldwide in 2017. The European Union, China, Turkey, the United States, and India are prominent tomato-producing nations. Out of the 160 million tons of tomatoes produced overall, about 40 million tons are processed [2]. Plant diseases can occur across the whole life cycle of crop plants and are one of the biggest threats to human welfare. Plant diseases result in a 13–22% yield loss and billions of dollars are lost in economic terms [3].

Nonetheless, the major constraints in tomato cultivation and reduced yield are due to the phytopathogens. The tomato plants fall susceptible to a variety of biotic and abiotic stresses from seedling to maturity stage [4]. The *Fusarium oxysporum* species are extremely harmful and widely distributed around the world. The Fusarium wilt disease of tomato caused by *Fusarium oxysporum* f. sp. *lycopersici* is a major pathological constraint affecting tomato crops worldwide. Fusarium wilt is one of the most common and destructive diseases, as the pathogen is soil-borne and can be transmitted through water and contaminated soil [5]. The pathogen can persist as chlamydospores in soil and agricultural leftovers for up to six years in the absence of a sensitive host [6]. Any stage of the crop can be easily infected by the *F. oxysporum*. The pathogen damages the vascular system of the plant and the noticeable symptoms can appear very late until the plant begins bearing fruits. This disease is still a major problem in tomato-growing areas due to the highly variable nature of the pathogen [7].

Beneficial endophytic microbiomes significantly influence plant responses under stressed conditions by mediating the functioning of the plant micro-ecosystem [8]. The biological management strategy for wilt diseases is a durable and economical approach without damaging the environment. The effectiveness of biological control agents can be sometimes comparable to the toxic chemical fungicides used to control wilt disease [9]. Biological control agents have been used for more than a century to ensure the long-term management of several important pathogens of field crops [10]. Different studies reported the use of Bacillus bacteria to manage plant diseases [11,12,13]. The ability to produce resistant endospores and antibiotics makes Bacillus an attractive biological control agent. These microbes are equipped with the production of antibiotics, lytic enzymes, and phytohormones, and can diminish the pathogen infection pressure by competitive exclusion and reducing the growth of pathogens without killing them [14,15]. Secondly, they cause resistance in plants against invading pathogens by the activation of induced systemic resistance [16].

To initiate these beneficial responses, specific signaling molecules are secreted by the microbes which are recognized by the plants [17,18]. Plants are equipped with pattern-recognition receptors (PRRs) used to sense elicitor chemicals (microbial-/pathogen-associated molecular patterns) produced by microbes. PRRs are mainly cell surface recognition proteins playing a vital role in the signaling process that initiates the plant’s immune responses [19]. LysM receptor kinase proteins are members of the PRRs family, responsible for the perception of signal molecules secreted by microbes [20,21].

Secondly, LysM domain proteins are responsible for the reception of the pathogen-associated molecular patterns (PAMPs) that trigger plant immune responses to avoid or limit the invasion of pathogens [22]. In Arabidopsis and rice plants, PAMPs secreted by the pathogens are perceived by LysM proteins [20,23]. LysM and chitin molecules form a heterotetramer complex activating the downstream immune responses [24,25]. Different members of LysM-type receptor kinases have been identified in tomato plants regarding their roles in signaling and symbiosis. Among them, SlLYK12 in tomato plants is mainly involved in microbial colonization [26]. This dual function of SlLYK12 receptor kinases in beneficial symbiosis and pathogen-triggered innate immunity makes them the target of choice in immuno- and bioinformatic studies.

Due to the use of diverse mechanisms by Bacillus bacterial to manage plant disease and the importance of receptor kinases in symbiosis and plant defense responses against invading pathogens, the present study aimed at the imperative use of the consortia of beneficial Bacillus microbes and synthetic chemical agonist capable to dock with LysM receptor kinases SlLYK12 of tomato to manage Fusarium wilt disease. Employing this integrated approach based on the bioassay-guided selection of biocontrol agents and searching synthetic agonists/chemicals by in-silico studies to facilitate symbiosis and trigger receptor kinases-mediated immune responses is likely to be a novel approach for combating plant disease.

## 2. Materials and Methods

### 2.1. Isolation of Rhizospheric Bacterial Strains

The heat shock method was used for the selective isolation of bacteria belonging to the Bacillus genera [19]. The rhizosphere soil samples were collected from healthy-looking vegetable crop plants in agricultural fields situated in different areas of the Punjab Province, Pakistan. The soil samples were collected by uprooting 3–5 healthy looking plants from each collection site (Table S1). The soil adhering to the plants root system was removed and pooled together to form one composite sample of rhizospheric soil from each collection site. The plant material and coarse roots were removed from the soil and remaining soil was used for isolation of bacterial microbes. For isolation purposes, one gram soil sample was mixed in distilled sterilized water (10 mL) and incubated at 80 °C for 10 min in the hot water bath [27]. The whole mixture was serially diluted up to 10^−7^ dilutions using distilled autoclaved water. Finally, 100 µL of the mixture from the last three dilutions was spread on Luria Bertani (LB) agar media plates (g/L: Yeast extract-5.0 g, Peptone-10 g, NaCl-10 g, Agar-15.0 g, pH 7). The plates were incubated at 30 ± 2 °C overnight. All single colonies were cultured on LB agar plates using the streaking inoculation method.

### 2.2. Selection of Antagonistic Bacterial Strains

Antagonistic bacteria were screened in-vitro on potato dextrose agar (PDA) medium plates in a dual-culture assay. Virulent culture of *F. oxysporum* f. sp. *lycopersici* isolate IAGS3 causing wilt disease in tomato was obtained from Fungal Biotechnology Laboratory, Department Plant Pathology, University of the Punjab, Pakistan. The 5 mm diameter plugs from one-week-old culture of *F. oxysporum* were transferred to one side of the media plates. The bacterial isolates were inoculated on the other side of the media plate in a straight-line manner. Each treatment was repeated three times. All plates were then kept at 28 °C for 5 days. Bacteria were characterized into three categories e.g., bacteria showing zone of growth inhibition; bacteria showing contact inhibition; bacteria showing no Inhibition [28]. The bacteria showing a clear zone of growth inhibition were selected for further studies.

### 2.3. Molecular Identification of Best-Performing Bacterial Strains

To identify the best-performing strains (IS1, IS6, and IS7), their 16S rDNA motif were amplified and obtained sequences were analyzed. Briefly, a pure culture of bacteria was grown in LB broth medium at 30 ± 2 °C, under continuous shaking (180 rpm) in a shaking incubator overnight. Total genomic DNA was extracted with the help of Rapid Bacterial Genomic DNA Isolation Kit (Sangon Biotech, Shanghai, China) according to the provided protocol. Afterward, routine PCR was performed to amplify the 16S rDNA sequence using the universal primers 27F (5′-AGAGTTTGATCCTGGCTCAG-3′) and 1492R (5′-GGTTACCTTGTTACGACTT-3′) [29]. After successful PCR, the obtained PCR products were sequenced by the Sanger dideoxy method at Macrogen Incorporation (Seoul, Republic of Korea). The Phylogenetic trees were constructed based on the 16S rRNA sequences with the MEGA version X software [30]. The maximum likelihood method [31] was adopted to construct the tree with 1000 bootstrap replications.

### 2.4. Screening of Potential Agonist/s of Tomato Receptor-Like Kinases SlLYK12

#### 2.4.1. Preparation of Protein Structure and Quality Analysis

Tomato receptor-like kinase SlLYK12 was chosen as the target protein because of its dual role in plant symbiosis and defense response [26,32,33,34]. Homology modeling of SlLYK12 was performed using Alphafold to generate a 3D structure from the protein sequence retrieved from the public database (NCBI Accession No. NP_001234725). The Ramachandran plot was generated and VERIFY3D, ERRAT, PROVE, and PROCHECK, criteria were used to analyze the overall quality of the model [35].

#### 2.4.2. Model Refinement and Validation

The selected protein model was repaired and refined by running the YASRARA md_refine macro provided in the YASARA Structure software, version 23.4.25 [36]. The macro runs a 500 ps simulation and saves snapshots every 25 ps. All parameters were kept at the values defined by the macro. This macro is responsible for energy minimization with combined steepest descent, fixing the backbone atoms to avoid potential damage to the model, and full unrestrained all-atom simulated annealing minimization. The refined model was further subjected to molecular dynamics simulations for >50 ns using the md_run.mcr macro of YASARA and AMBER14 force-field. The structure of the protein was simulated in a rectangular box with periodic boundaries and filled with a water density of 0.997 g/mL. Berendsen barostat and thermostat were used to control the temperatures and pressures during simulations. The ion-concentration was kept at 0.9% NaCl.

Afterward, the protein model was analyzed and validated using the ProtParam tool of ExPASy Proteomics Server for various parameters such as estimated half-life, theoretical pI, instability index, aliphatic index, and grand average of hydropathicity (GRAVY) [37].

#### 2.4.3. Virtual Screening by Molecular Docking

The database of phytochemicals was downloaded from the Plant Secondary Compound Database http://pscdb.appsbio.utalca.cl/viewIndex/index.php (accessed on 13 April 2023). These included alkaloids, flavonoids, phenylpropanoids, and phytohormones. Before docking, ligands were prepared to perform the following: conversions of 2D to 3D, energy minimization, the addition of hydrogen atoms, and necessary structural optimizations using the Openbebal program. Similarly, the receptor molecule was prepared by adding polar hydrogen atoms and removing water molecules on Discovery Studio software, version 2019. The molecular docking studies of active compounds were performed using AutoDock Vina by PyRx virtual screening software, version 0.8 [38]. A blind docking strategy was adopted because a completely new receptor molecule was used with no previous knowledge of potential binding sites for ligands [39,40,41]. Preliminary docking was performed to screen the whole legend library and separate the legend/s fulfilling the selection criteria. The results for receptor-ligand complexes were arranged in ascending energy order to determine the interaction profile. Ligand/s showing maximum vina score and commercial availability were chosen. Afterward, re-docking of the selected legend was performed. The selected conformation of the top order ligand/s associated with SlLYK12 receptor kinases was analyzed to dissect the interaction profiles by Discovery Studio software version 2019 and Pylip online server available at https://plip-tool.biotec.tu-dresden.de/plip-web/plip/index (accessed on 29 May 2023). PyMOL was used as molecular visualization software, version 2.5.0.

### 2.5. In-Vivo Effect of the Synthetic Elicitor and Consortium of Bacillus Strains on Fusarium Wilt Disease Development and Growth of Tomato Plants

The combined application of biotic and synthetic elicitor was performed by soil drenching and foliar application, respectively. Before seed priming, antagonistic bacteria were analyzed for consortia compatibility by growing them in a single petri plate as streaked as the cross pattern. For soil drenching, an aqueous suspension of the consortia bacterial strains was made. For this, all the bacteria strains (*Bacillus velezensis* BS6, *Bacillus thuringiensis* strain BS7, *Bacillus fortis* strain BS9) were raised in 250 mL flasks, separately, containing LB broth (g/L: Yeast extract-5.0 g, Peptone-10 g, NaCl-10 g, pH 7) medium under shaking (150 rpm) conditions at 30 ± 2 °C overnight and cells were collected by centrifugation. The next day, the cultures were centrifuged at 10,000 rpm for 5 min, and the pellets washed with distilled autoclaved water. Pallets were resuspended in distilled autoclaved water to reach the OD of 1 at 600 nm to achieve a concentration of 10^8^ cfu/mL. A combined inoculum was prepared by mixing equal volumes of the aqueous suspensions of the three bacterial isolates to develop consortium suspension [42]. Plastic pots were filled with sterilized potting mix and fifty mL of consortium suspension was added in the allotted pots. The next day, surface sterilized tomato seeds were cultivated in the pots. After emergence, thinning was performed to ensure uniformity of seedlings, and one healthy seedling was left in the pot. Additionally, a chemical elicitor (quercetin) was applied as a foliar application at varying concentrations (0.01, 0.1, 1.0 mM) twenty days post-emergence, according to the experimental design as described in Table 1. Ten days after quercetin application, conidial suspension of FOL was prepared as suggested by PURWATI and Hidayah [43]. Briefly, the culture of *F. oxysporum* was grown in potato dextrose agar (PDA) medium and incubated at 30 ± 2 °C for seven days. The fungal conidia were removed by gentle scratching in the presence of distilled sterilized water using sterilized spatula and sieved by sterilized nylon cloth to remove debris. The concentration of conidial suspension was estimated by hemocytometer and diluted with the appropriate amount of sterile water to obtain desired density (≃10^6^ conidia/mL) of the conidial suspension. Fifty mL of conidial suspension was added to the soil adjacent to the roots by drenching. Plants receiving FOL served as pathogen controls. Plants raised from only distilled autoclaved water-primed seeds and sprayed with distilled autoclaved water served as non-treated controls. The fungicide control consisted of the soil drenching of Carbendazim fungicide (1.5 g/L), as suggested by [44].

Five biological replicate pots were included in each treatment and the whole experiment was repeated twice. Plants were irrigated with distilled sterilized water and kept at green house under natural daylight conditions. After twenty days of pathogen application, the disease scoring was performed as described by Shanmugam, et al. [45] (0 = no symptoms; 1 ≤ 25% of the leaves with symptoms; 2 = 26–50% of the leaves with symptoms; 3 = 51–75% of the leaves with symptoms; and 4 = 76–100% of the leaves with symptoms). The disease index (DI) was determined using the below-mentioned formula [46]:

DI = [Σ rating × number of plants rated)/Total number of plants × highest rating] × 100

Additionally, growth attributes of tomato plants like shoot length, root length, shoot biomass, and root biomass were also determined at harvest. Total chlorophyll contents were analyzed by spectrophotometer, as described by Arnon [47].

### 2.6. Analysis of the Biochemical Basis of Induced Defense Responses in Tomato Plants against Fusarium Wilt Disease

Another independent pot experiment was performed using the same strategy mentioned in the previous section using selected treatments but with a shorter duration. Selection of treatments was based on the outcome of the previous experiment, as the combined application of Quercetin (1.0 mM) + Consortium provided maximum suppression of Fusarium wilt disease. Hence, the treatments included in this experiment were as following: untreated control, pathogen control, quercetin (1.0 mM) + FOL, consortia + FOL and consortia + quercetin (1.0 mM) + FOL. After five days of pathogen application, the harvest was taken.

#### 2.6.1. Analysis of Total Phenolics and Plant Defense-Related Enzymes

The quantification of total phenolic compounds and enzymes involved in phenylpropanoid pathways was performed five days after pathogen application. For the extraction of total phenolic compounds, 5 g of leaf material was grinded using liquid nitrogen and extracted in 50 mL of 95% ethyl alcohol for 20 min at 60 °C. The extract was filtered using paper filters and the final volume was adjusted to 50 mL by adding distilled water. The total phenolic compounds were analyzed by the Folin–Ciocalteu method [48]. Briefly, a 0.5 mL aliquot from the extract was mixed with the 2.5 mL Folin–Ciocalteu’s reagent solution (0.2 M) and 2 mL of 7.5% sodium carbonate. The reaction mixture was incubated for 15 min at 40 °C. The OD was measured at 760 nm.

The leave samples were ground into a fine powder using liquid nitrogen and enzyme extraction was performed in 0.1 M sodium phosphate buffer (pH 7.5) containing 1 mM EDTA and 1% PVP in an ice bath. After centrifuging at 3550× *g* for 10 min at 4 °C, the supernatant was separated and used as the enzyme extract. Briefly, for the quantification of peroxidase (PO) activity, the reaction mixture contained 10 mM guaiacol, 0.1 mL of the enzyme extract, 120 mM H_2_O_2_ inside 50 mM phosphate buffer (pH 5.5). The formation of tetraguaiacol was monitored at 470 nm [49]. For the quantification of polyphenol oxidase (PPO), the reaction mixture consisted of 0.1 mL enzyme extract, 20 mM catechol inside 100 mM of phosphate buffer (pH 5.5). The whole mixture incubated for 10 min at 37 °C. The reaction was stopped by adding 1.22 M trichloroacetic acid (2.0 mL) and OD was noted at 525 nm [50]. The phenylalanine ammonia-lyase (PAL) activity was examined using a method from [51]. The reaction mixture contained 100 mM Tris- HCl, 40 mM l-phenylalanine, and 0.1 mL of the enzyme in a total volume of 1 mL at pH = 8.8. The reaction was performed out at 37 °C for 30 min and terminated by adding 50 µL HCl (4 M).

#### 2.6.2. Analysis of the Metabolomic Profile of Tomato Plants

A non-targeted metabolomic analysis was performed on the UHPLC triple quadrupole MS/MS apparatus (Agilent Technologies, Santa Clara, CA, USA) to observe the changes in the metabolomic profile of the tomato plants. For that purpose, leaves from the plants of different treatments were ground to powder material in the pestle and mortar using liquid nitrogen. The mixture of (methanol/water, 80/20, *v*/*v*) containing 1 ng/µL of the internal standard was used for extraction [52]. The extract was centrifuged and passed by a microfilter assembly. Chromatographic separation was performed on an Agilent 1200 ultra-performance liquid chromatography system (Agilent Technologies, Santa Clara, CA, USA) fitted with a C18 analytical column (Agilent Technologies, Santa Clara, CA, USA). The data for the identification and quantification of compounds were obtained from a Triple Quad tandem mass spectrometer (6470) system equipped with an electrospray ionization source (ESI). A QC sample was made after pooling the samples of all treatments in a single vial in equal quantities. The mobile phase A consisted of 0.1% formic acid (*v*/*v*) in deionized water and mobile phase B consisted of 0.1% formic acid (*v*/*v*) in methanol. The following gradient conditions were adopted [53]: 95% A and 5% B for the first 5 min, solvent A decreased to 45% and B increased to 55% up to 22 min, solvent A 5% and B 95% over the course of 3 min and remained unchanged for one minute, solvent A 95% and B 5% for 3 min until the end of the run.

The MS scan range was 50–1500 *m*/*z* with a 100 ms scan time. The acquired mass data were converted into an mzxml format. Afterward, the data were loaded onto MZmine 2.53 software for qualitative and quantitative analysis [54,55]. Identification of compounds was performed using the NIST MS/MS library and the previously published literature [56,57].

### 2.7. Statistical Analysis

All the experiments were repeated twice and mean values are provided. The data were analyzed statistically. The analysis of ANOVA and DNMRT was performed by using the Excel addon “DSAASTAT” [58].

## 3. Results

### 3.1. Selection of Antagonistic Rhizospheric Bacterial Strains

**Figure 1:**
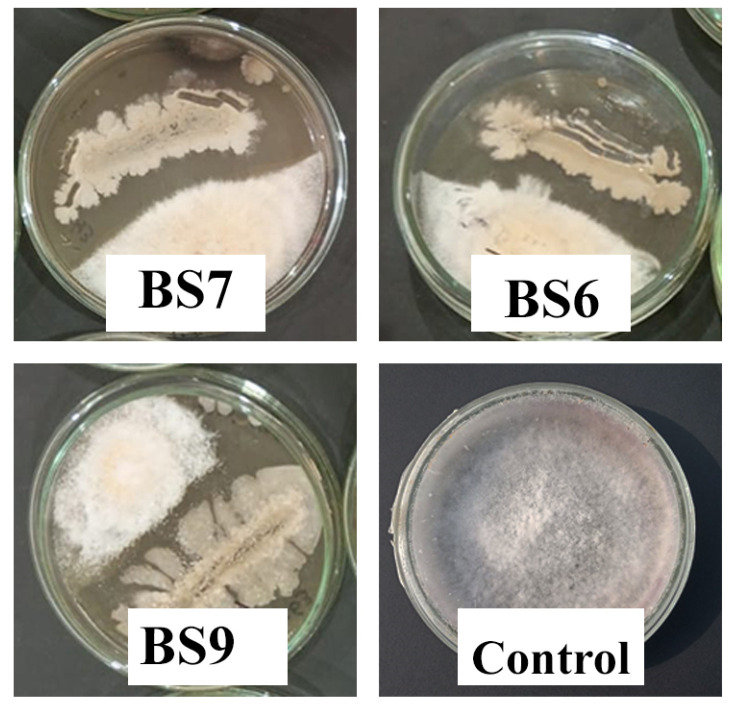
In-vitro analysis of the antagonistic properties of Bacillus isolates. Clear zones of growth inhibition were developed by strains BS7, BS6, and BS9.

Altogether, 11 different bacterial isolates were isolated from the rhizosphere of tomato plants using the heat shock method aimed at Bacillus genera. The purified bacterial isolates were screened for the presence of antagonistic activity against FOL using a dual culture assay. The analysis showed that out of the 11 isolates, 5 could successfully inhibit the growth of Fusarium wilt pathogen (Table 2). In these cases, three bacterial isolates (BS6, BS7, and BS9) showing a clear zone of inhibition were separated (Figure 1; Table 2). Two of the bacterial isolates could inhibit the growth of the pathogen upon contact with a fungal pathogen colony. These were denoted as bacterial isolates having contact inhibition activity. The rest of the bacterial strains showed no inhibition activity (Table 2). Bacteria showing a clear zone of inhibition were selected for further experimentation.

### 3.2. Molecular Identification of Bacterial Strains

**Figure 2:**
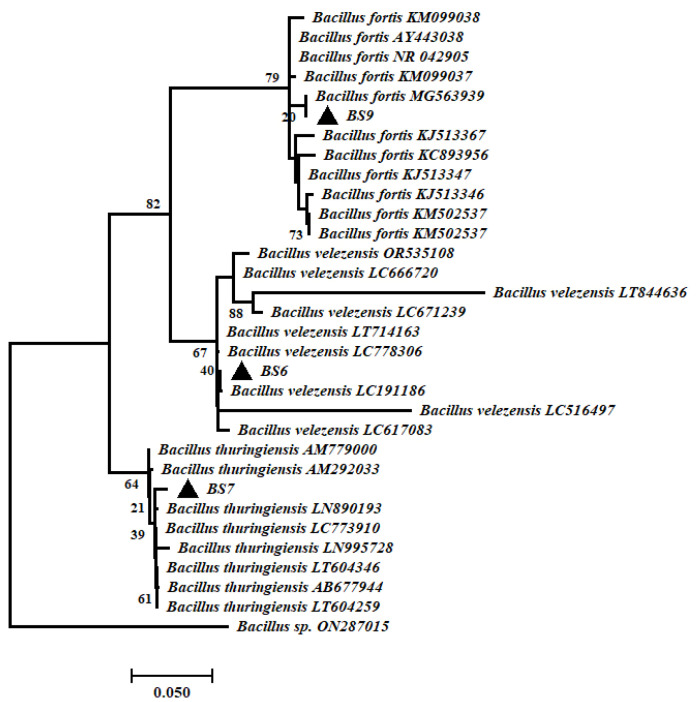
Maximum likelihood tree of sequences of selected bacterial strains and retrieved from NCBI database. Numbers at nodes represent the percentage values given by 1000 bootstrap analysis samples. Scale represents evolutionary lineages changing over time.

Three bacterial strains with antagonistic activity against FOL were identified by the amplification and sequencing of the 16rRNA region. The BLAST analysis showed that the bacterial isolates BS6, BS7, and BS9 shared >99% homology with *Bacillus velezensis* (NCBI Accession No. LC191186), *Bacillus thuringiensis* (NCBI Accession No. LT838123), and *Bacillus fortis* (NCBI Accession No. MG563939), respectively (Figure 2). The obtained sequences were submitted in NCBI database under accession numbers OR645476, OR645552, and OR645575. After performing the BLAST analysis for homology, the sequences together with their closest relatives in NCBI GenBank were retrieved and used to construct a maximum likelihood phylogenetic tree (Figure 2).

### 3.3. Screening of Potential Agonist/s of Tomato Receptor-Like Kinases SlLYK12

Before the molecular docking, the SlLYK12 model was refined and the quality analysis of the refined SlLYK12 protein was performed by online quality structure assessment tools (Table 3). Here, no residue of the SlLYK12 was in the disallowed region, whereas >90% of residues were found in the favored region (Table 3). The quality analysis using VERIFY3D, ERRAT, and PROCHECK servers indicated a good overall quality of the SlLYK12 protein model. The refined SlLYK12 model was further analyzed for physicochemical parameters such as theoretical isoelectric point, estimated half-life, instability index, aliphatic index, and the grand average hydropathicity (Table S2). The half-life of SlLYK12 (30 h), the instability index (<40), the higher aliphatic index (87.62), and the lower GRAVY value (−0.05) of our refined SlLYK12 model present the good quality of the receptor representing the stable receptor model (Table S2).

**Figure 3:**
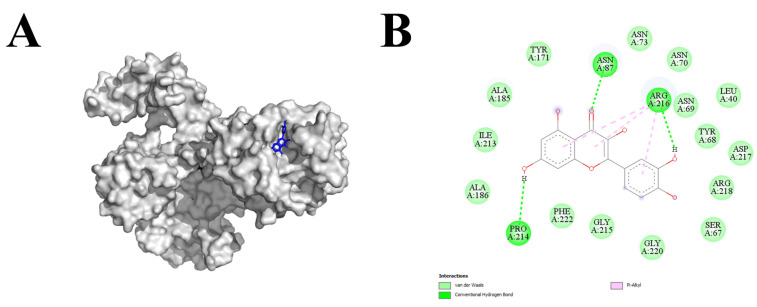
The snapshots of quercetin ligand docked within the SILKY12 receptor (**A**) and their interactions (**B**).

The molecular docking analysis was performed using a library of phytochemicals carefully developed keeping in view the commercial availability and economic feasibility. The top score legends are mentioned in Table 4 with binding affinity >8 after performing a preliminary docking analysis. Quercetin (8.7 binding affinity) was selected based on the top highest affinity scores and devised screening criteria to avoid a lengthy study. The SlLYK12 + quercetin complex comprised three hydrogen bonds and the rest of the intermolecular bond e.g., van der Waals force, pi alkyl (Figure 3) as depicted by the Discovery Studio.

### 3.4. In-Vivo Effect of the Synthetic Elicitor and Consortium of Bacillus Strains on Fusarium Wilt Disease Development and Growth of Tomato Plants

**Figure 4:**
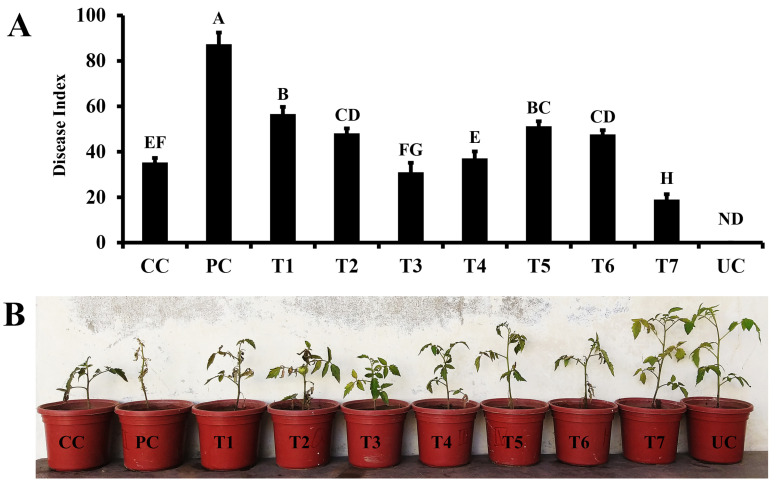
The potential of bacterial consortia and synthetic elicitor to suppress Fusarium wilt disease. Vertical bars represent standard error. (**A**) Fusarium wilt disease index analysis of tomato plants. (**B**) Plants showing the effect of different treatments on the development of Fusarium wilt disease. Capital letters present the level of significance by ANOVA and DNMRT at *p* = 0.05. details of treatments are provided in Table 1.

In pot trials, different treatments consisting of synthetic elicitor (quercetin) and bacterial consortia in different combinations were assessed for their efficiency in suppressing fungal wilt disease. A varied degree of protection was observed ranging 17–69% under greenhouse conditions. The highest level of disease index was observed in the pathogen control treatment. The valuation of Fusarium wilt protection showed that the combination of both synthetic elicitor (quercetin) and bacterial consortia was significantly higher than disease protection provided by the quercetin or bacterial consortia individually (Figure 4). A combination of quercetin (1.0 mM) and bacterial consortia reduced the disease index up to 69.63% as compared to the pathogen control (Figure 4). Similarly, another treatment consisting of the combined applications of quercetin (0.1 mM) and bacterial consortia reduced the disease index up to 51.36% compared to the pathogen control. The foliar application of quercetin alone significantly reduced disease index up to 29.27% (0.01 mM) and 37.61% (0.1 mM) over pathogen control. Treatment with bacterial consortia alone reduced disease by >35% compared to the pathogen control (Figure 4).

Regarding growth parameters, the minimum shoot length, root length, shoot biomass, root biomass, and chlorophyll contents were related to the pathogen control plants, followed by carbendazim fungicide control (Table 5). The rest of the treatments significantly increased the aforementioned growth attributes of tomato plants compared to the pathogen control (Table 5). The combined application of quercetin (1.0 mM) and bacterial consortia significantly increased shoot (1.6- fold) and root biomasses (2.4- fold) compared to pathogen control plants, respectively. These outcomes made it evident that the combined treatment of quercetin (1.0 mM) and bacterial consortia effectively rescued the growth of tomato plants attacked by the Fusarium wilt pathogen and made it comparable to the non-treated control plants in some instances (Table 5). Hence, based on the findings of pot trials the treatment consisting of the combined application of quercetin (1.0 mM) and bacterial consortia was selected to elucidate the possible mechanisms behind disease suppression. It is to be mentioned here that the rest of the treatments including consortia alone also significantly increased the growth attributes of tomato plants but the application of quercetin combined with the consortia showed a more pronounced increase in the growth attributes of tomato plants.

### 3.5. Analysis of the Biochemical Basis of Induced Defense Responses in Tomato Plants against Fusarium Wilt Disease

#### 3.5.1. Analysis of Total Phenolics and Defense-Related Enzymes

Now the efforts were diverted to determine the role of quercetin (1.0 mM) and bacterial consortia in the induction of tomato systemic resistance through the comparative quantification of enzymes involved in phenylpropanoid pathways and phenolic compounds.

The results found that a combination of quercetin (1.0 mM) and bacterial consortia had a higher significant effect on the inducible production of total phenolics, and activities of PO, PPO, and PAL enzymes than those of single elicitor treatments (Table 6). The combined applications have increased to 1.42-, 1.79-, 2.36- and 1.34-fold increase in the quantities of total phenolics, PO, PPO, and PAL enzymes compared to the pathogen control (Table 6). The outcomes further confirm that the treatment of quercetin and consortia alone are potential inducers in defense responses, but to a lesser extent compared to the combined application of quercetin and bacterial consortia (Table 6).

#### 3.5.2. Non-Targeted Metabolomic Analysis

**Figure 5:**
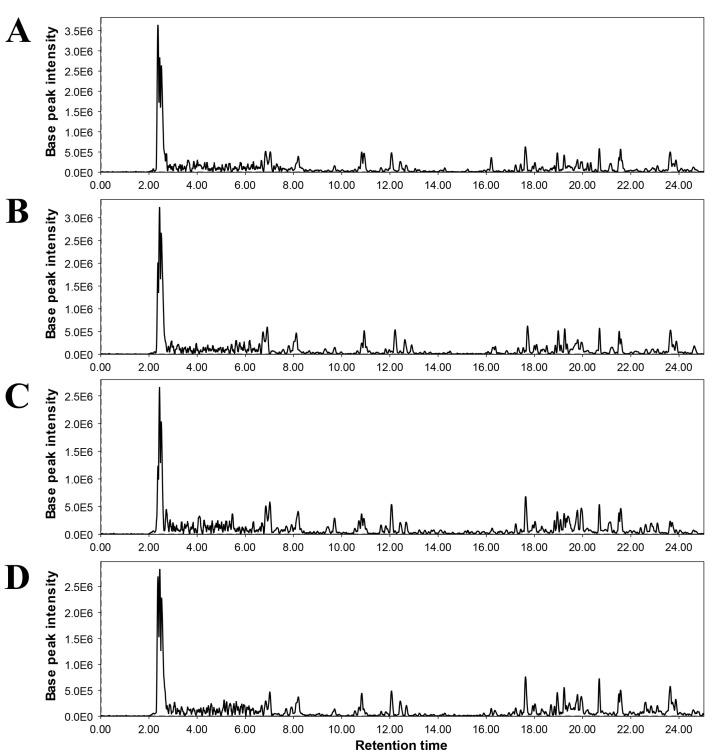
Representative chromatograms of non-targeted metabolomic analysis of tomato plants under different treatments. (**A**) = pathogen control, (**B**) = quercetin (1.0 mM) + FOL, (**C**) = consortia + FOL, and (**D**) = consortia + quercetin (1.0 mM) + FOL.

**Figure 6:**
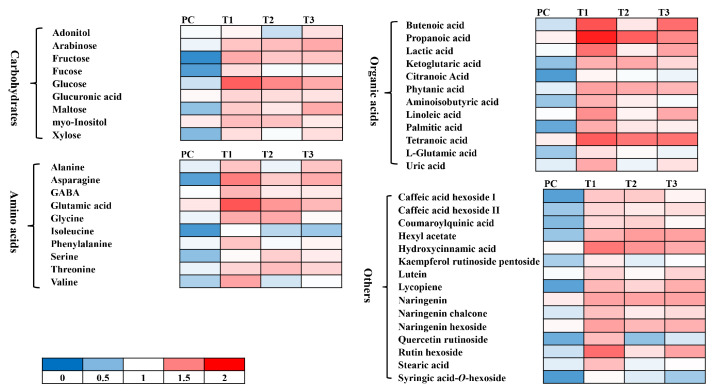
Heat map showing changes in the levels of different metabolites. Data are presented as fold change as compared to the untreated control treatment.

**Figure 7:**
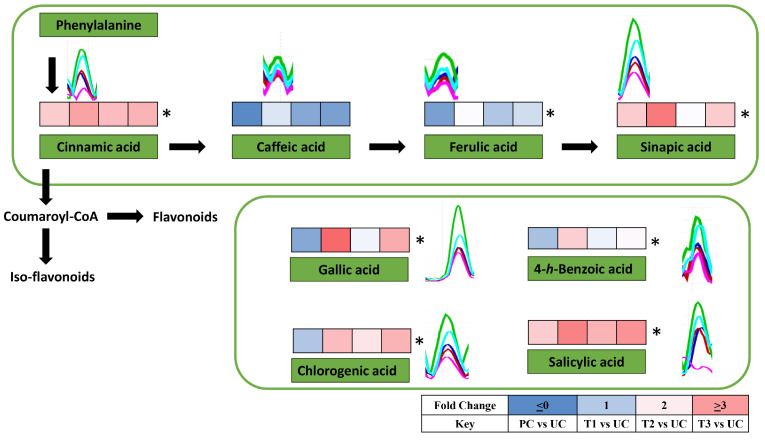
Heat map showing changes in the levels of different phenolic acids. Data are presented as fold change as compared to the untreated control treatment. * Represent significant differences among treatments as governed by ANOVA at *p* > 0.05.

The up-regulated defense system in tomato plants was further analyzed by performing non-targeted metabolomic analysis. The metabolomic data were obtained from UHPLC-triple quadrupole -MS/MS analysis. The representative chromatograms indicate varying levels of different metabolites among different treatments (Figure 5). The quantities of phenylpropanoids including ferulic acid, cinnamic acid, caffeic acid, and gallic acid, positively responded to the quercetin (1.0 mM) and bacterial consortia in either combination (Figure 6 and Figure 7). Most of the phenylpropanoid metabolites showed higher values of fold change in combined treatment (quercetin 1.0 mM + bacterial consortia) compared to the rest of the treatments (Figure 6).

The rest of the metabolites interfering with plant physiology belonged to carbohydrates, amino acids, alkaloids, and organic acids, etc. (Figure 6). The tomato plants exhibited a more evident physiological response to the combined application of quercetin 1.0 mM + bacterial consortia followed by the Fusarium wilt pathogen. The metabolic response was also significant for the rest of the treatments (Figure 6). For pathogens alone, the effect on major classes of compounds was clear compared to that of non-treated control plants. Similarly, the metabolomics responses varied clearly in tomato plants treated with quercetin and bacterial consortia alone compared to the non-treated control plants (Figure 6).

## 4. Discussion

Tomato is a valuable vegetable crop in the Solanaceae family. Tomatoes are grown both in open fields and in greenhouses. This crop generates a high return for farmers and provides plenty of job opportunities for rural residents [59]. Rhizospheric beneficial bacteria have several strategies, both direct and indirect, to manage plant diseases [60]. In this study, an imperative approach was used based on the consortia of antagonistic Bacillus microbes and synthetic elicitors capable of triggering symbiosis and plant immunity, to manage Fusarium wilt disease of tomato plants.

The plant disease suppression mediated by the *Bacillus* spp. rely on the direct antagonistic effects of antimicrobial metabolites produced by bacteria against pathogens or by activation of defense responses in the host plant [61]. In the first phase of the present study, we isolated Bacillus isolates using the heat shock method from the rhizospheric soil and demonstrated their antagonistic capabilities in in-vitro studies. The strains were differentiated into non-antagonists, antagonists with contact inhibition, and antagonists with the clear zone of inhibition. The three Bacillus isolates (BS6, BS7, and BS9) showed a clear zone of inhibition against the radial growth of *F. oxysporum*. The isolates were identified as *Bacillus velezensis* BS6, *B. thuringiensis* BS7, and *B. fortis* BS9 based on 16s rRNA gene sequencing. These findings are in line with some previous studies that Bacillus species, including *Bacillus velezensis*, *B. thuringiensis*, and *B. fortis* strains have antagonistic activities against different plant pathogens [62,63,64,65,66,67]. These antagonistic properties can be attributed to the production of antifungal lipopeptides by Bacillus species with antifungal activities for different phytopathogenic fungi, including *F. oxysporum* [68]. The findings of this preliminary study provide us with a basis to develop consortia of these antagonistic Bacillus isolates for the management of Fusarium wilt during subsequent experiments.

Synthetic elicitors are the molecules that can induce plant immune responses upon interaction with the respective plant receptors. Synthetic elicitors may trigger defense reactions by mimicking the function of natural elicitors or signaling molecules [69]. Exogenous application of synthetic elicitors e.g., salicylic acid and other benzoic acid derivatives, such as acetylsalicylic acid, have been reported to induce resistance responses of tobacco plants against diseases caused by viruses by triggering the production of pathogenesis related proteins [70]. Receptor-like kinases play important roles in plant immunity. Plants use a large number of receptor kinases as pattern recognition receptors for the detection of microbial-derived molecular patterns and/or synthetic elicitors to initiate inducible defense [71].

A target-based approach aims at identifying chemicals that selectively interfere with a defined target. This has been successfully used in the pharmaceutical research but there is a dearth of studies aimed at screening synthetic compounds to search for novel compounds to interface with a defined targets in plants. To this end, in-silico studies were performed to find synthetic agonists capable of binding with the receptor-like kinases “SILKY12” of tomato plants. The homology modeling is used to obtain a 3D representation of the target receptor whenever experimental structures are not available for docking. The use of DeepMind’s artificial intelligence model, AlphaFold (AF), set a milestone within the field of homology modeling [72]. The SlLYK12 model was created with the Alphafold, online server. The model showed the superior quality/criteria scores devised by the modeling servers [37]. Further quality analysis of the selected models from each modeling server was performed by online quality structure assessment tools available on the Saves server. The quality analysis using VERIFY3D, ERRAT, and PROCHECK servers also indicated a good overall quality of the trRosetta model. Henceforth, validation scores suggest that trRosetta-modelled SlLYK12 can be used for further molecular docking analysis. The refined trRosetta model was further analyzed for physicochemical parameters, such as theoretical isoelectric point, estimated half-life, instability index, aliphatic index, and the grand average hydropathicity. Protein-ligand blind docking strategy was adopted as it is considered a powerful method to explore the best binding sites of receptor molecules and the binding conformation of ligands. Among five top-score legends, quercetin was selected based on the top highest affinity scores and screening criteria to avoid a lengthy study. The SlLYK12 + quercetin complex was stabilized by the presence of three hydrogen bonds and the rest of the intermolecular bond e.g., van der Waals force, and hydrophobic contacts. The presence and number of hydrogen bonding indicated that the agonist could trigger the SlLYK12 receptor kinase to modulate further downstream processes [73]. Similarly, the presence of hydrophobic contacts indicates stable protein folding, biological activeness, and reduced undesirable interactions [74].

Ultimately, the treatments for pot trials were devised based on the findings of these preliminary studies. The consortia capability of three strains was analyzed before the pot trial. Afterward, consortia were applied as soil drenching and quercetin as a foliar amendment. Here, lower concentrations of quercetin were used (0.01, 0.1 and 1.0) to ensure the economic feasibility of the whole process. The results of pot trials showed that the combined use of consortia of Bacillus microbes in association with the quercetin (1.0 mM) proved to be an effective strategy to manage Fusarium wilt disease of tomato as well as increased plant growth. The combined treatment based on consortium and quercetin reduced the disease index by >60% and significantly improved plant growth attributes including shoot length, root length, shoot biomass, root biomass, and total chlorophyll contents in tomato plants.

Previously, different studies have focused on the use of microbial consortia for disease control. From the microorganism’s perspective, this is mainly based on the synergistic or additive effects and aims to achieve a higher pest or disease control than their components. The findings of the pot trial were found to be consistent with some previous studies that showed the combined use of beneficial microbes along with the synthetic elicitor can provide better protection against both biotic and abiotic stress conditions [75,76]. Wei et al. [77] reported that the consortium of biocontrol agents effectively suppressed the clubroot disease better than the application of a single strains. Similarly, Srivastava et al. [78] reported that the use of the consortia of beneficial fungi and bacterial microbes significantly enhanced tomato yield and suppressed the Fusarium wilt disease incidence. The application of Bacillus consortia can produce multiple antibiotic substances [79], which can play a critical role in the suppression of the pathogen population inside plant rhizosphere.

Quercetin facilitates several plant physiological processes, such as seed germination, antioxidant machinery, and photosynthesis, as well as induces proper plant growth and development [80]. In this study, the effect of exogenous application of quercetin on tomato plants was seen to suppress disease, promote growth, and stimulate the physiological processes of tomato. Some previous studies have successfully demonstrated the beneficial effect of quercetin on cowpea [81] and tomato plants [82] under drought and salt stress, respectively. As well as the beneficial effect of the exogenous application of quercetin on plant growth, chlorophyll content has also been confirmed in previous studies conducted on tomato [83] and wheat plants [84]. In addition, the application of quercetin can enhance the symbiotic beneficial impact of consortia and can further rescue plant growth under disease stress conditions. As observed in the pot trial, the integration of Bacillus consortium (soil drenching) alongside quercetin (foliar spray) greatly improved the growth-related attributes compared to the treatment where both were applied alone, possibly due to the collaboration with phytohormones synthesis, increased cell division, and better performance of photosynthesis machinery.

A thorough understanding of the interactions between tomato plants, Bacillus consortium, and quercetin having an effective impact on plant disease suppression and development is required. To this end, another independent experiment was performed to elucidate the mechanisms behind disease suppression mediated by the combined application of a consortium of Bacillus strains and quercetin. Consequently, it was observed that the combined approach was more stimulatory for increasing the production of total phenolic compounds and enzymes involved in the phenylpropanoid pathway like peroxidase, polyphenol oxidase, and phenylalanine ammonia-lyase than the Bacillus consortium or quercetin alone. Peroxidase is an important defense protein that contributes to the biosynthesis of lignin [85] and antimicrobial phytoalexins inside plants. Similarly, polyphenol oxidase and phenylalanine ammonia-lyase play an important role in the biosynthesis of toxic substances via the phenylpropanoid pathway to hinder the growth of pathogens inside plants [86]. In a previous study, induced resistance was obtained through increased production of defense-related enzymes in tomato plants treated with rhizospheric microbes and chemical elicitor salicylic acid [87]. Their findings proved that upon pathogen inoculation, relatively higher activities of defense-related enzymes were seen treated with both bacterial and chemical elicitors compared to the plants only receiving pathogen inoculum.

A non-targeted metabolomic analysis was performed to further improve our understanding to this end. The findings were comparable as mentioned in the previous section. A large number of perturbations were seen in the metabolic profile of tomato plants under different treatments. It could be due to the fact that Bacillus consortium in combination with quercetin greatly affected the physiology of plants, as the varying abundance of a range of metabolites including carbohydrates, amino acids, organic acids, phenylpropanoids, polyols, flavonoids, etc., was seen across different treatments. Surprisingly, the metabolic reprogramming in tomato plants exposed to the pathogen and Bacillus consortium+ quercetin was more extensive compared to the other treatments. The representative chromatograms and heatmap showed vividly that combined treatment influenced the metabolites in tomato plants differently under the attack of a pathogen from the treatments where Bacillus consortium and quercetin were applied alone. The pathogen attack negatively affected the production of a range of metabolites with few exceptions., The application of Bacillus consortium and quercetin either alone and/or in combination seemed to restore the production of several metabolites in tomato plants. The application of the Bacillus consortium and quercetin increased the production of different phenolic acids in tomato plants subsequently challenged with the pathogen. These changes in the levels of phenylpropanoids may affect the ability of the tomato plant to restrict the growth and colonization of pathogens, reflected in a lowered disease index in pot trials [88]. Similarly, increased production of carbohydrates, amino acids, sugars, and polyols could be attributed to the higher availability of raw material for building blocks in tomato plants, leading to increases in growth-related parameters [89]. These findings are of great importance to understanding a complex mechanism governing disease suppression and growth enhancement under the influence of beneficial microbes and synthetic elicitors. Additional omics can also be used to further understand the response of plants to non-toxic chemicals and beneficial microbes in the presence of a nasty pathogen.

## 5. Conclusions

We have demonstrated that the imperative use of Bacillus consortium and quercetin application suppresses the Fusarium wilt disease of tomato and significantly enhances tomato defense responses and growth parameters. In the presence of beneficial microbes, quercetin can act as a cofactor to ensure symbiosis, elicit plant defense responses, and regulate physiological traits. The quercetin can be used as an additive in the beneficial microbes-based formulation to facilitate symbiosis and trigger receptor kinases-mediated immune responses. In conclusion, our findings provide practical information for the imperative use of beneficial microbes and chemical elicitors to manage plant disease.

## Figures and Tables

**Table 1:** Details of treatments.

Treatment	Description
CC	Carbendazim control
PC	Pathogen control (FOL)
T1	Quercetin (0.01 mM) + FOL
T2	Quercetin (0.1 mM) + FOL
T3	Quercetin (1.0 mM) + FOL
T4	Consortium + FOL
T5	Quercetin (0.01 mM) + Consortium + FOL
T6	Quercetin (0.1 mM) + Consortium + FOL
T7	Quercetin (1.0 mM) + Consortium + FOL
UC	Untreated control

**Table 2:** Characterization of bacterial strains for antagonism properties.

No	Code	Gram Staining	Antagonistic Phenotype
1	BS1	Positive	NA
2	BS2	Positive	CGI
3	BS3	Positive	NA
4	BS4	Positive	NA
5	BS5	Positive	NA
6	BS6	Positive	ZGI
7	BS7	Positive	ZGI
8	BS8	Positive	CGI
9	BS9	Positive	ZGI
10	BS10	Positive	NA
11	BS11	Positive	NA

NA = No Activity, CGI = Contact growth inhibition, ZGI = Zone of growth inhibition.

**Table 3:** Quality assessment of the predicted 3D models.

Parameter	Value
PROCHECK	
Errors	5
Warning	2
Pass	2
ERRAT	97.03
Ramachandran plot	
FR	91.8
AR	8.1
GR	0.2
DR	0.00
Verify3D	62.4

FR = most favored regions, AR = additional allowed regions, GR = generously allowed regions, DR = disallowed regions.

**Table 4:** Estimate of the overall binding free energies of the top five legends.

Compound	Formula	Molecular Weight	Binding Affinity (kcal/mol)
Quercetin	C_15_H_10_O_7_	302.2	−8.7
Galbacin	C_20_H_20_O_5_	340.4	−8.7
Traumatin	C_12_H_20_O_3_	212.2	−8.3
7-Oxotyphasterol	C_28_H_48_O_5_	464.7	−8.2

**Table 5:** Effect of the application of consortium of antagonistic bacterial strains and quercetin on growth parameters of tomato plants.

Treatment	Shoot Length (cm)	Root Length (cm)	Shoot Biomass (g)	Root Biomass (g)	Total Chlorophyll (mg/g fw)
CC	08.01 ± 0.5 e–g	08.62 ± 0.5 ef	1.82 ± 0.07 de	0.31 ± 0.02 gh	11.23 ± 0.76 e–g
PC	09.28 ± 0.8 ef	07.23 ± 0.5 e–g	1.58 ± 0.09 ef	0.24 ± 0.02 i	07.17 ± 0.04 h
T1	11.12 ± 0.9 de	09.26 ± 0.5 de	2.06 ± 0.08 b–d	0.43 ± 0.03 ef	10.87 ± 0.76 fg
T2	10.38 ± 1.4 de	10.71 ± 0.7 cd	1.93 ± 0.12 cd	0.51 ± 0.04 c–e	15.91 ± 1.27 cd
T3	13.66 ± 1.5 cd	11.96 ± 0.7 b–d	2.24 ± 0.13 bc	0.57 ± 0.02 cd	16.08 ± 1.27 cd
T4	14.26 ± 1.3 b–d	12.81 ± 0.9 bc	2.59 ± 0.14 bc	0.65 ± 0.03 c	16.53 ± 0.98 c
T5	17.05 ± 1.0 bc	12.54 ± 1.1 bc	2.66 ± 0.24 ab	0.59 ± 0.04 cd	19.58 ± 1.13 a
T6	19.17 ± 1.5 ab	15.97 ± 1.4 ab	2.89 ± 0.10 a	0.81 ± 0.05 ab	18.32 ± 1.64 ab
T7	21.09 ± 1.1 a	17.26 ± 1.2 a	3.07 ± 0.18 a	0.87 ± 0.06 a	19.53 ± 1.25 a
Con	23.18 ± 1.6 a	15.62 ± 1.1 ab	3.24 ± 0.21 a	0.92 ± 0.05 a	21.35 ± 1.57 a

Values are mentioned as mean ± standard error. Letters present a level of significance as governed by ANOVA and DNMRT at *p =* 0.05. Details of treatments are provided in Table 1.

**Table 6:** Effect of combined application of rhizobacteria and synthetic elicitors on defense-related activities of tomato plants against Fusarium wilt disease.

Treatment	Total Phenolics (mg g^−1^ FW)	PO (ΔOD min^−1^ g^−1^ FW)	PPO (ΔOD min^−1^ g^−1^ FW)	PAL (ΔOD min^−1^ g^−1^ FW)
Untreated Control	2.03 ± 0.14 ^e^	0.09 ± 0.00 ^e^	0.72 ± 0.03 ^c^	0.18 ± 0.01 ^d^
Pathogen Control	4.93 ± 0.51 ^b–d^	0.68 ± 0.04 ^c^	1.12 ± 0.25 ^b^	0.49 ± 0.02 ^bc^
Consortia + Quercetin (1.0 mM) + FOL	7.18 ± 0.37 ^a^	1.03 ± 0.25 ^a^	2.68 ± 0.09 ^a^	0.66 ± 0.05 ^a^
Consortia + FOL	5.39 ± 0.20 ^bc^	0.87 ± 0.03 ^b^	2.17 ± 0.15 ^a^	0.52 ± 0.03 ^ab^
Quercetin (1.0 mM) + FOL	6.28 ± 0.37 ^ab^	0.56 ± 0.02 ^cd^	1.32 ± 0.07 ^b^	0.63 ± 0.04 ^a^

Values are mentioned as mean ± standard error. Letters present a level of significance as governed by ANOVA and DNMRT at *p =* 0.05.

## Data Availability

The data supporting the findings of this study are available within the article and Supplementary Materials.

## Supplementary Materials

The following supporting information can be downloaded at: https://www.mdpi.com/article/10.3390/microorganisms11102603/s1, Table S1: Details of the collection sites of Rhizospheric soil; Table S2: Physiological parameters of the receptor.
