# Supplementary material for: The Imperative Use of Bacillus Consortium and Quercetin Contributes to Suppress Fusarium Wilt Disease by Direct Antagonism and Induced Resistance
Source: Microorganisms. 2023 Oct 21;11(10):2603. doi: 10.3390/microorganisms11102603 (PMC10609423; doi:10.3390/microorganisms11102603)
Supplement: Supplementary file 1 [file microorganisms-11-02603-s001.zip › microorganisms-2649284-supplementary.pdf]

Table S1: Details of the collection sites of Rhizospheric soil.

No.	Province	City/Town	Coordinates
1	Punjab	Lahore	31.4790° N, 74.2662° E
2	Punjab	Lahore	31.4220° N, 74.1739° E
3	Punjab	Kasur	31.0249° N, 73.8479° E
4	Punjab	Kasur	30.9906° N, 74.2708° E
5	Punjab	Bahawalpur	29.4249° N, 71.7637° E
6	Punjab	Raheem yar khan	28.3688° N, 70.2422° E

Table S2: Physiological parameters of the receptor.

Parameter	Value
Estimated half-life	30 hours
Theoretical pI	5.78
Instability index	33.35
Aliphatic index	87.62
GRAVY	-0.050
